# Effect of Drying Temperature on Physical, Chemical, and Antioxidant Properties of Ginger Oil Loaded Gelatin-Sodium Alginate Edible Films

**DOI:** 10.3390/membranes12090862

**Published:** 2022-09-06

**Authors:** Ahmed Al-Harrasi, Saurabh Bhatia, Mohammed Said Al-Azri, Sana Ullah, Asim Najmi, Mohammed Albratty, Abdulkarim M. Meraya, Syam Mohan, Mohammed F. Aldawsari

**Affiliations:** 1 Natural and Medical Sciences Research Center, University of Nizwa, P.O. Box 33, Birkat Al Mauz, Nizwa 616, Oman; 2 School of Health Science, University of Petroleum and Energy Studies, Prem Nagar, Dehradun 248007, India; 3 Department of Pharmaceutical Chemistry and Pharmacognosy, College of Pharmacy, Jazan University, P.O. Box 114, Jazan 45142, Saudi Arabia; 4 Pharmacy Practice Research Unit, Department of Clinical Pharmacy, College of Pharmacy, Jazan University, P.O. Box 114, Jazan 45124, Saudi Arabia; 5 Substance Abuse and Toxicology Research Center, Jazan University, Jazan 45142, Saudi Arabia; 6 College of Pharmacy, Prince Sattam Bin Abdul Aziz University, P.O. Box 173, Al-Kharj 11942, Saudi Arabia

**Keywords:** edible films, gelatin, antioxidant, sodium alginate, drying temperature

## Abstract

The drying temperature is one of the crucial parameters that impacts the physical, chemical, and biological properties of edible films (EFs). This parameter determines the degree of crystallinity, which can further impact the film’s mechanical, barrier, and optical properties. The present work is designed to investigate the effect of different drying temperature conditions (25 °C and 45 °C) on ginger essential oil (GEO) loaded Gelatin-sodium alginate composite films over their physical, chemical, and antioxidant properties. Results indicated that drying of films at 25 °C had a positive effect on certain properties of the EFs, such as the moisture content (MC), water solubility (S), swelling degree (SD), water vapor permeability (WVP), and mechanical and optical properties. SEM analysis showed that films dried at 25 °C presented more uniform surface properties with fewer cracks and pores compared to films dried at 45 °C. TGA analysis demonstrated the higher thermal stability of the films when dried at 25 °C. Findings obtained from X-ray diffraction (XRD) and fourier-transform infrared spectroscopy (FTIR) showed film crystallinity and electrostatic interactions between GE, SA, and GEO. Results obtained from antioxidant assays revealed that films dried at 25 °C showed comparable antioxidant capacity to that of butylated hydroxytoluene (BHT). Furthermore, it was found that the addition of SA and GEO to the blank GE films improved their physical, chemical, and antioxidant properties. The present work suggests that GEO loaded GE-SA based films showed better physical, chemical, and antioxidant potential when dried at a lower temperature. These novel materials can be utilized as potential packaging materials in the food industry.

## 1. Introduction

The food packaging industry is currently emphasizing more on the development of eco-friendly, biodegradable, safe and efficient packaging materials as the current petrochemical-based packaging materials have unfavorable effects on human health as well as the surrounding environment. Chemicals present in plastic packaging materials including phthalates, bisphenol A or polybrominated diphenyl ethers, can leach into food products, resulting in their ill effects on human health. Thus, to mitigate the unwanted effects of conventionally used packaging materials, more focus has been given to the development of alternative packaging materials in the form of edible films, which are safe, nontoxic, biocompatible, have high end consumer acceptability, and can effectively improve the shelf life of the food.

Process parameters such as pH, temperature, homogenization speed, viscosity of the film forming solution, drying temperature, preconditioning of edible films, casting material, etc., are the crucial parameters that need to be investigated with respect to specific polymeric composite films or individual polymer-based films. The drying temperature impacts their degree of reorganization or crystallinity, which can cause variation in the barrier, mechanical, and optical properties of the EFs. This process parameter is considered one of the most crucial steps during the development of EFs as an inadequate drying environment can result in films with flaws, e.g., cracks, pores, blisters, more roughness, and warping [1]. Thus, the effects of drying conditions, specifically temperature range and duration of exposure to EFs, must be investigated. Some of the major changes have been observed during the drying of EFs, such as crystallization, from rubbery to glassy stage, and phase separation due to thermodynamic incompatibility. Interaction as well as assembly among different components at the molecular level have been reported. These changes are attributed to drying induced by solvent evaporation as well as migration of solute [2]. Additionally, previous reports in the literature suggested that changes in drying temperature significantly impact the rate of solvent evaporation as well as solute immigration, which can ultimately influence the physical as well as chemical properties of the films. Thus, changes in the duration of drying significantly control the physical and chemical attributes of the films [3].

Gelatin (GE), a hydrophilic biodegradable polymer, has been extensively used as a food packaging material because of its safety profile, excellent gas barrier, and film-forming properties [4]. However, individual gelatin-based packaging materials always face problems in terms of their unsatisfactory mechanical and optical properties with high brittleness. Recent trends in food packaging composite materials indicate that films of two or three polymers can be used to overcome the challenges faced by single polymers. Until now, limited information is available on gelatin/sodium alginate-based composite films [4]. Thus, blending gelatin with a food-grade, safe, biodegradable polysaccharide such as sodium alginate could be an excellent approach. Sodium alginate demonstrates excellent film-forming properties, and its films present high transparency and mechanical strength. On the other hand, sodium alginate (anionic polysaccharide) and gelatin, (cationic proteinogenic polymer and its charge depends on the pH and type of gelatin) due to their opposite charges and show high electrostatic interaction against each other to form a stable polyelectrolyte complex. Furthermore, their hydrophilic nature makes them more compatible with each other while mixing their respective film forming solutions. However, in order to reduce the water affinity, it is crucial to impart a hydrophobic character by adding any natural nontoxic food grade hydrophobic material. This hydrophobic material ideally must have the ability to improve the physical and chemical properties of the composite material and also provide protection against detrimental oxidation and microbial contamination [5]. Ginger oil has been known for its high antioxidant, antimicrobial as well as aromatic properties and is considered as ″generally recognized safe″ (GRAS) by the Food and Drug Administration (FDA), USA [6]. Incorporation of sodium alginate and GEO could be a better strategy to overcome the challenges faced by gelatin and drying temperature. Considering this approach, the current study was designed to study the effects of different drying temperatures (25 °C and 45 °C) on the the structural, chemical, and antioxidant properties of GEO incorporated into gelatin-sodium alginate films. Findings obtained from this investigation could be utilized to further understand the behavior of GE-SA films and molecular interactions between GEO, gelatin, and sodium alginate at different drying conditions.

## 2. Materials and Methods

### 2.1. Chemicals

Gelatin powder Special ex. Porcine (Type B) and sodium alginate (food grade) were purchased from Sisco Research Laboratories Pvt Ltd., Andheri, India. Tween 80 (polysorbate) was purchased from Merck KgaA, Gernsheim, Germany. Glycerol was purchased from BDH Laboratory Supplies, Dorset, UK. Ginger oil (100 mL) (Batch No.: NNIGIEO/104/0821) was supplied by Nature Natural India, Uttar Pradesh, India. Folin and Ciocalteu’s phenol (FCP) reagent, 2,2-diphenyl-1-picrylhydrazyl (DPPH), and butylated hydroxytoluene were purchased from Sigma-Aldrich, Taufkirchen, Germany.

### 2.2. Preparation of the Films

For the fabrication of edible films, gelatin powder (8 g) was dissolved in distilled water (200 mL) by using a magnetic stirrer (Daihan Scientific, Seoul, Korea) at 500 rpm followed by heating at 45 °C for 30 min. The resultant solution was equally divided into 6 parts and labeled F1–F6. SA (3% *w*/*v*) was added to F5 and F6 solutions. As mentioned in Table 1, glycerol (1.5% *w*/*w* based on gelatin content) was added as a plasticizer to the F1–F6 solutions and subjected to mixing at 500 rpm for 10 min. Subsequently, GEO (1% *w*/*w* gelatin) and the emulsifier Tween 80 (1.0% *w*/*v*) were added to F3–F6. All solutions (F1–F6) were incubated and agitated at 50 °C for 15 min. Then all the film forming solutions were poured into polystyrene petri plates (height: 15.7 mm, diameter: 90 mm). To study the effect of the change in drying temperature on the films, F1, F3, and F5 were subjected to drying at 25 °C for 5 h while F2, F4, and F6 were subjected to drying at 45 °C for 5 h using a horizontal flow oven (Daihan Scientific, Seoul, Korea). After drying, all film edges were treated with 70% ethanol for 5–10 min and peeled off from the plastic petri plate surface. This treatment eases the peeling of the films from the petri plate surface and reduces the chances of their breakage during the process. Films obtained were kept in a desiccator at 25 °C and 50 ± 4% RH for 72 h for further study.

### 2.3. SEM (Scanning Electron Microscopy) Analysis

The surface and cross-sectional structural features of the EFs were assessed by using SEM (JSM6510LA, Analytical SEM, Jeol, Japan) at 20 kV. The F1–F6 films were coated with gold prior to taking images. Liquid nitrogen was used to fracture the F1–F6 films manually and affixed over the aluminum ends by using tape. They were then coated with a thin layer of gold.

### 2.4. Thermal Stability Assessment

The thermal gravimetric (TG) analyzer (TA Instruments, New Castle, DE 19720, USA, SDT-Q600) was used to assess the thermal stability of the samples. Samples were analysed in a nitrogen atmosphere and scanned in the range of 25–600 °C with a heating rate of 10 °C/min.

### 2.5. Analysis of FTIR Spectra

An FTIR Spectrometer (InfraRed Bruker Tensor 37, Ettlingen, Germany) was used to determine the elemental structure of the samples by setting them up with an attenuated total reflection (horizontal) device (45° ZnSe). For each spectrum, 32 scans in the range of 400–4000 cm^−1^ with a resolution of 4 cm^−1^ were performed. All the measurements were conducted at room temperature.

### 2.6. X-ray Diffraction (XRD) Studies

The crystalline structure of the samples was determined by an XR-Diffractometer (Bruker D-8 Discover, at 40 kV with 2θ ranging from 5° to 50°).

### 2.7. Thickness

A micrometer (Mitutoyo Micrometer 2046F, Kanagawa, Japan) was used to determine the thickness (in mm) of the fabricated samples.

### 2.8. Mechanical Testing

A texture analyzer (Stable Micro Systems Ltd., Godalming, UK) was used to assess the mechanical properties, including elongation at break (EB), Young modulus (Ym) and tensile strength (TS), of the samples. The ASTM 93 D882-2010 standard procedure was followed for the mechanical assessment of the films [7]. Films were sliced into rectangular-shaped strips of 20 mm × 50 mm. EFs were examined at a mechanical crosshead speed of 50 mm per min at an initial grip separation of 30 mm. The measurements were performed in triplicate and then the average value was calculated. The TS (MPa) and EB (%) were computed as follows:

TS = Mxf/A(1)

where maximum force at break (N) is indicated by Mxf, and the area of the EF cross-section (m × m) is represented by A.

The following Equation (2) was used for the calculation of elongation at break (EB):

EB = (D1 − D2)/D2 × 100%(2)

where EB indicates elongation at break (%), D2 represents the length of the film at the time when it breaks down (mm). D1 was the original length of the EF.

### 2.9. Water Vapor Permeability (WVP)

The procedure that was used in our previous work with slight changes was utilized to find out the WVP [8,9]. The WVP of the samples was measured as per the following equation:

P = (ΔW × S)/(A2 × T × ΔWP)(3)

where P = WVP (×10^−12^ g cm cm^2^ s^−1^ Pa^−1^); ΔW = variation in weight of flask; S = mean film thickness (cm); A2 = film area (cm^2^); T = time (s), ΔWP = variation in water vapor partial pressure (atm) between both sides of the EFs.

### 2.10. Swelling Degree (SD) and Water Solubility (WS)

WS and SD were determined using the Romero et al. method with slight modifications [10]. Samples (F1–F6) were sliced into 20  ×  60  mm slices and then the initial weight of the EFs measured followed by drying using a desiccator to achieve a preliminary dry weight. Subsequently, the sample obtained was placed into water (25 mL) and maintained at room temperature for 24  h to ease the swelling. Afterwards, the samples were subjected to drying and their corresponding weight was measured. To determine the ultimate dry weight of the samples, EFs were dried until a steady weight was achieved at 65 °C. WS as well as SD were measured as per the following equation:

WS = (initial dry weight − final dry weight)/initial dry weight × 100(4)

SD = (wet weight − initial dry weight)/initial dry weight × 100(5)

where WS stands for water solubility (%) and SD stands for swelling degree (%).

### 2.11. Moisture Content Analysis

The moisture content of the samples (F1–F6) of size 2 cm × 2 cm was measured by placing them in a petri dish. Subsequently, these petri dishes made up of glass samples were subjected to drying using a hot air oven for 12 h at 105 °C. Films were dried till a steady dry weight was attained and the difference in weight was calculated by using the following Equation (6):

MC = (WT_1_ − WT_2_)/WT_2_ (6)

where in this equation, moisture content is abbreviated as MC, preliminary film mass (mg) was abbreviated as WT_1_, and final mass was abbreviated as WT_2_.

### 2.12. Oxygen Barrier Property

Iodometric titration was used to investigate the oxygen permeability of films. The oxygen barrier capacity of the fabricated films can be linked to the peroxidation of camellia oil, which can be assessed by the reducing agent (sodium thiosulphate) using the methods by Kurt et al. and Zhou et al. [11,12] with minor changes as mentioned in our previous work [9]. The oxygen barrier property (OB) was determined by using the following equation:

OB (g/100 g) = [(A−B) × C × 0.1269]/M × 100(7)

where A represents the quantity of standard Na_2_S_2_O_3_ solution (mL) utilized by the EFs, and B signifies the amount of standard Na_2_S_2_O_3_ solution (mL) consumed by the blank. C signifies the standard sodium thiosulphate (mol/L) concentration, 0.1269 is the value of iodine equal to one milliliter of standard Na_2_S_2_O_3_ solution (g), M represents the quality of the sample (g), and 100 denotes the conversion factor.

### 2.13. Antioxidant Assays

#### 2.13.1. Sample Preparation

Samples were prepared as per the method used previously by Ruiz et al. [13]. Edible films (500 mg) were weighed initially and then added to methanol (15 mL). Resultant solutions were subjected to vortex mixing (VWR Int., Darmstadt, Germany). At 10 °C, mixed samples were centrifuged at 10,000 RPM for 10 min. F1–F7 supernatant solutions were tested for antioxidant activity (F1–F6 are test samples, while F7 is the standard solution of butylated hydroxytoluene).

#### 2.13.2. Total Phenolic Content Assay

The determination of total phenolic content was done as per the previous procedure with a few modifications [14]. In this procedure, resultant supernatant solutions (F1–F7) were allowed to mix with a desired quantity of Folin and Ciocalteu’s reagent. Then the solution was subjected to incubation at 25 °C with continuous mixing, followed by treatment with an equivalent amount of sodium bicarbonate. The absorbance of the solution was measured using a microplate reader at 517 nm. The findings gained were stated as mg/gm of GAE (gallic acid equivalent). Absorbance was determined in triplicate.

#### 2.13.3. DPPH Assay

EFs were assessed for their ability to scavenge DPPH radicals using an earlier method [15]. The resultant supernatant solutions (F1–F7) were allowed to mix with an equivalent amount of DPPH solution using 96-well microplates. The solution obtained was subjected to incubation (30 min) at 37 °C with continuous mixing. A microplate reader was used to measure the absorbance using a blank preparation at 517 nm. BHT was utilized as a standard. The procedure was repeated three times and the mean was obtained. The percentage of inhibition was evaluated as per the following Equation (8):

Percentage inhibition (%) = (ABc − ABt)/(ABc) × 100(8)

where ABc = control solution absorbance ABt = test solution absorbance.

#### 2.13.4. ABTS Assay

The antioxidant potency of the sample was evaluated by scavenging the 2,2-azinobis-(3-ethylbenzothiazoline-6-sulfonic acid (ABTS+) using the method provided by Re R. et al. (1999) [16]. Stock solutions of the samples (EFs) were made, and 10-μL amount from each sample was transferred to a 96-well assay plate. After the addition of ABTS + solution of EFs samples or Trolox standards, the absorbance was taken at 734 nm. A blank reading of all solvents was run in each assay and subtracted from the final reading. The assay was performed in triplicate along with standards.

### 2.14. Statistical Analysis

The mean values plus standard deviation (S.D.) of the three independently performed replicates are represented for all results. For testing the significance of variations amongst mean values at 5%, one-way analysis of variance followed by Duncan’s test was performed using statistical analysis software.

## 3. Results and Discussion

### 3.1. Scanning Electron Microscopy (SEM)

**Figure 1:**
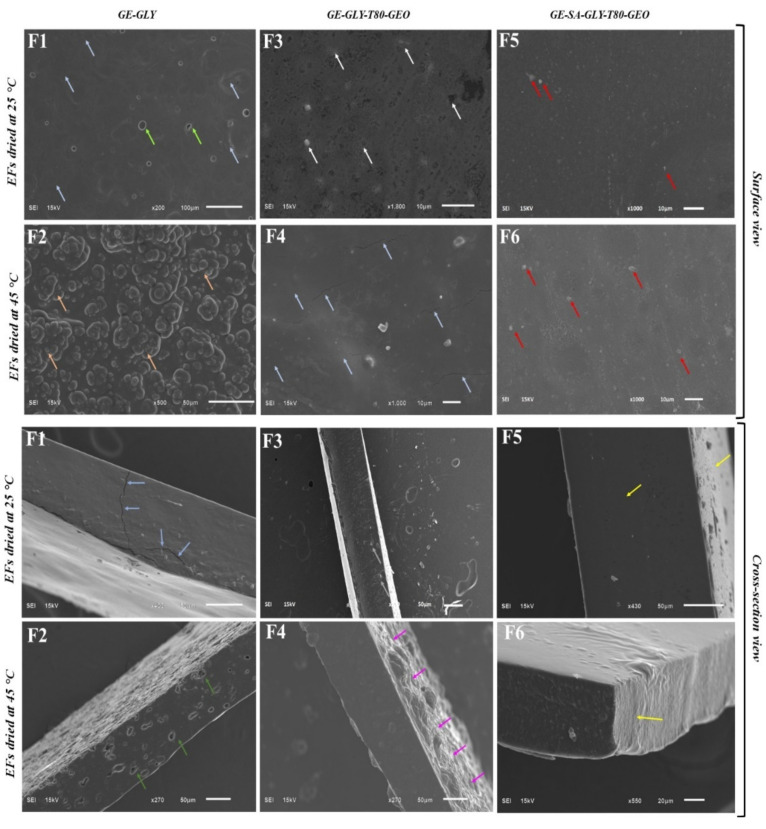
SEM images (both surface and cross-section) of GE based film with and without SA. (**F1**): GE-Gly, dried at 25 °C, (**F2**): GE-Gly, dried at 45 °C, (**F3**): GE-Gly-T80-GEO, dried at 25 °C, (**F4**): GE-Gly-T80-GEO, dried at 45 °C, (**F5**): GE-SA-Gly-T80-GEO, dried at 25 °C, (**F6**): GE-SA-Gly-T80-GEO, dried at 45 °C. The blue color indicates the cracks, orange color represents surface roughness, white color shows tricuspid shaped cracks, red color represents particles at the surface, deep green color indicates pores, pink color represents surface bulges, and yellow color indicates smoothness at cross-sectional level.

Surface and cross-sectional morphological assessment of blank (F1, F2) and composite (F3–F6) gelatin-based edible films was carried out using scanning electron microscopy (SEM). The drying temperature as well as the addition of SA and GEO have considerably influenced the physical architecture of GE-based blank EFs [17]. Composite films exhibited more favorable morphology (surface and cross-sectional) compared to blank GE films. The microstructure of the blank films at a higher resolution showed multiple cracks, roughness, and more particles at the surface (Figure 1). Previous studies report that the existence of breaks and agglomerates in the film matrix can affect its physical properties [18]. Current findings reveal that the composite films dried at lower temperatures exhibited favorable characteristics. These films showed more uniformity and compactness when compared to other films. The overall homogenous surface morphology in the composite films could be attributed to the even distribution of oil and better compatibility between GE and SA. GEO loaded films showed less prominent micro-voids and a denser internal structure compared to blank films. The dense shape in the composite films reveals the formation of a strong cross-linking network between GEO and gelatin-sodium alginate. Previously, Wu, et al. [19] found that the addition of essential oil to films makes it more compact and smoother compared to blank films.

The current findings suggest that drying temperature has a considerable impact on the morphology of both blank and composite films. Films dried at 25 °C presented a more uniform surface and were found as more compact compared to films dried at 45 °C (Figure 1). The current findings were not in agreement with a previous study [3] in which different results were obtained at a higher temperature. As per an earlier study, the surface homogeneity at lower temperatures could be due to the presence of additional material that may have enhanced the cohesiveness of the matrix [20].

### 3.2. Thermal Analysis

**Figure 2:**
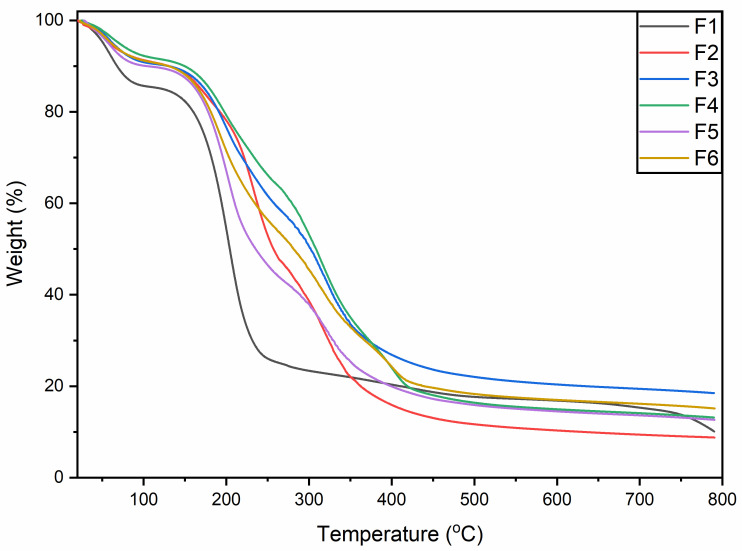
Thermogravimetric analysis (TGA) spectra of blank (F1, F2) and composite films (F3–F6). The first drop in weight loss (about or below 100 °C) is driven by the water molecule, the second drop after 150 °C is showing decomposition of the organic matter. F1 has highest moisture content and showed largest drop. (F1); GE-Gly, dried at 25 °C, (F2); GE-Gly, dried at 45 °C, (F3); GE-Gly-T80-GEO, dried at 25 °C, (F4); GE-Gly-T80-GEO, dried at 45 °C, (F5); GE-SA-Gly-T80-GEO, dried at 25 °C, (F6); GE-SA-Gly-T80-GEO, dried at 45 °C.

The thermogravimetric analysis (TGA) was performed to evaluate the thermal stability of the films (Figure 2). The weight loss occurred in three successive stages. The first stage of thermal decomposition/weight loss was from around 40 to 110 °C, which is associated with the evaporation of water [21]. Around 10% weight loss occurred during this stage in all samples except (F1), which underwent comparatively sharp weight loss compared to the rest of the preparations. This behavior of the films could be due to the addition of GEO or GE or both. Perhaps the addition of GEO or GE or both leads to this variation in the films compared to the blank film, F1 [20].

Major thermal decomposition was observed around 150–300 °C. This could be attributed to the thermal decomposition of the film components. As previously reported, the breakdown of glycerol-rich content occurs between 150 °C and 260 °C [22], whereas thermal decomposition of gelatin was reported at around 225 °C [23]. Overall, about 55% of the weight loss was observed at this stage. The thermal decomposition patterns of the blank film F1 were found to be sharper compared to the other films. The current TGA findings reveal that the addition of SA and GEO to the GE-based films increases the thermal stability. This phenomenon could be attributed to the better molecular interaction of film ingredients in the composite films. Edible film synthesis at a lower temperature has key advantages over films dried at a higher temperature. It has been reported that films dried at low temperatures (25 °C) maintain a denser polymeric network as well as moisture content, which leads to an increase in TS and a decrease in EB [24]. The carbonaceous residues of the films degrade at temperatures ranging from 300 to 415 °C [25]. At this stage, about 35% weight loss was observed and similar TGA patterns were followed by the films as at the previous stage (stage 2).

### 3.3. FTIR Data Analysis

**Figure 3:**
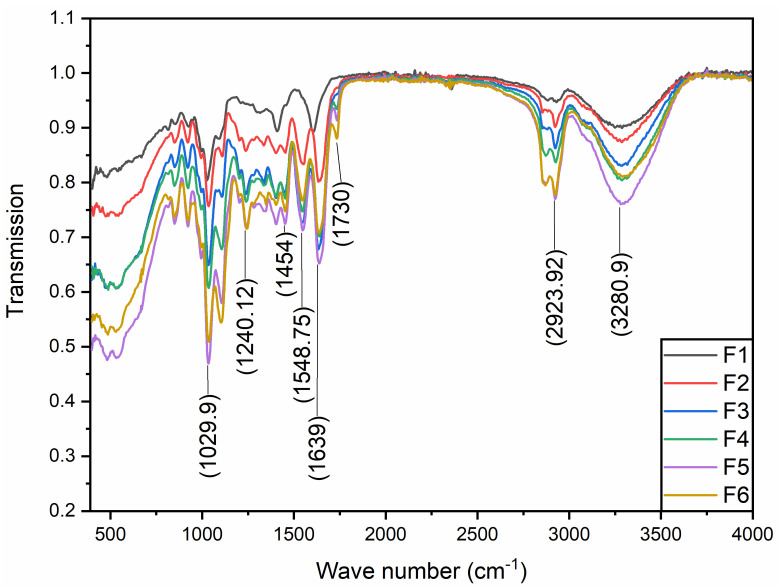
Representative FTIR spectra of blank (F1, F2) and composite films (F3–F6). (F1); GE-Gly, dried at 25 °C, (F2); GE-Gly, dried at 45 °C, (F3); GE-Gly-T80-GEO, dried at 25 °C, (F4); GE-Gly-T80-GEO, dried at 45 °C, (F5); GE-SA-Gly-T80-GEO, dried at 25 °C, (F6); GE-SA-Gly-T80-GEO, dried at 45 °C.

FTIR spectroscopy was performed to check the functional groups and chemical interactions between the gelatin, sodium alginate, and GEO. Moreover, this chemical assessment was utilized to draw a relationship between drying temperature and chemical interactions among the different components of the film (Figure 3). Since polymeric matrices of GE and GEs are enriched with amino, hydroxyl, and carboxylic functional groups, it is important to assess their chemical interaction among them as well as with phenolic components present in GEO [26]. Figure 3 shows the absorbance patterns of GE-based films dried at different temperatures. Overall, most of the FTIR band patterns of all the samples are similar (however, transmission intensity is different in all) except F1. Drying temperature has no significant impact on shifting band patterns but at the same time, it has an impact on shifting the wavenumber from lower to higher or higher to lower. This means that drying temperature as well as the addition of SA and GEO are impacting the chemical interaction of the film components.

Characteristic peaks reported at 3280, 1639, 1548.75, and 1240.12 cm^−1^ represent amide-A and free water, amide-I, amide-II, and amide-III of gelatin respectively [27]. Amide-A at 3280 cm^−1^ represents NH-stretching combined with hydrogen bonding; amide-I arises from the medium bending of the N-H group. The amide-II arises from the stretching vibration of C-N groups and the bending vibration of N-H groups; and the amide-III relates to the strong stretching of C-O or medium stretching of C-N groups [28,29]. Characteristic peaks reported at 2923.92, 1730.05, and 1454.24 cm^−1^ could be ascribed to the amide-B, carboxylic group, and carbonyl groups. The peaks between 1100 and 900 cm^−1^ could be attributed to carbonyl and sulfoxide groups [29]. FTIR band at 2840.85 cm^−1^ position, showed the vibration mode of C-C-H group; while 1730.05 cm^−1^ represents strong C=O stretching of aldehyde group; and FTIR band at 1454.24 cm^−1^ represents the medium bending of C-H group [29]. In addition, the peak recorded at 1035.71 cm^−1^ could be due to the possible interaction between glycerol’s OH group and the film structure. The FTIR bands at about 924 cm^−1^ indicate the crystalline structure of sodium alginate [30]. In the FTIR findings the characteristic peaks of GE-SA were found to be predominant, as in previously reported studies. An increase/decrease in the absorbance pattern might be due to a difference in the drying temperature and or to the addition of GEO [30].

FTIR analysis of the edible films concluded that composite film F5, dried at 25 °C, was found to be better than others because of the better bond strength and intensity observed from the transmission pattern. Higher absorbance or low transmission values were recorded for F5, followed by F6, F4, and F3 respectively, which reveals comparatively greater bonding and stronger molecular interaction between the functional groups in the given samples. Low transmission or higher absorbance pattern indicates more chemical interaction in a given sample and vice versa [31]. The addition of SA and GEO to blank films has had a prominent impact on the peak intensities and shift in wavenumbers at some positions, from lower values to higher values (Figure 3). FTIR spectra demonstrated that blank films behaved differently at different temperatures; a shift in band pattern was observed at different positions (1000–1600 cm^−1^). However, similar band patterns were observed in the composite films (F2–F6), which signifies no significant chemical shift among the different components of the films due to different drying temperatures. Thus, the different drying temperatures have less or no impact on the alteration of band pattern (in the case of wavenumber) but have changed the transmission pattern of the films.

### 3.4. X-ray Diffraction (XRD) Analysis

**Figure 4:**
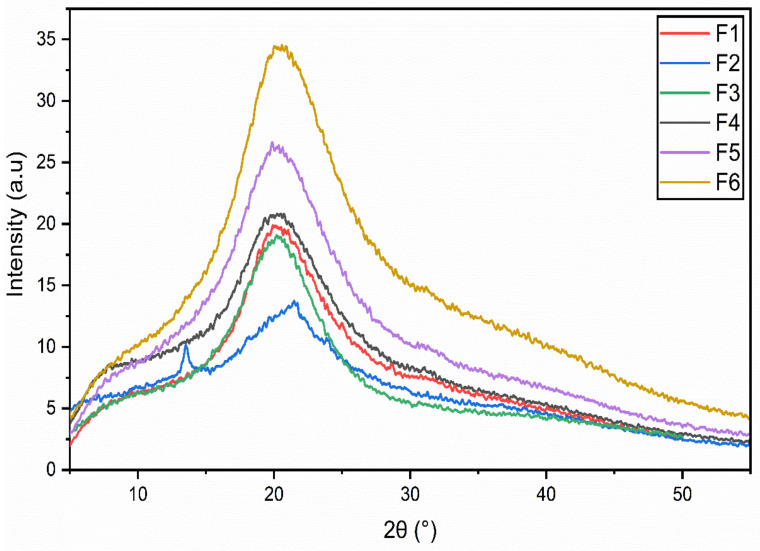
X-Rays diffraction (XRD) of blank (F1,F2) and composite films (F3–F6). (F1); GE-Gly, dried at 25 °C, (F2); GE-Gly, dried at 45 °C, (F3); GE-Gly-T80-GEO, dried at 25 °C, (F4); GE-Gly-T80-GEO, dried at 45 °C, (F5); GE-SA-Gly-T80-GEO, dried at 25 °C, (F6); GE-SA-Gly-T80-GEO, dried at 45 °C.

X-ray diffraction (XRD) was performed to accurately analyze the crystalline structures of the edible films. In all the edible films, a broader peak is observed at 20° of 2θ except for F2, which showed two peaks, one small and sharp peak at 13.5° and the second one at 21.4° of 2θ (Figure 4). The current XRD results are in agreement with earlier findings in which a broader peak was observed at 21° (2θ) [32].

It has been observed that the addition of sodium alginate and increased drying temperature (45 °C) significantly impacted the XRD patterns in terms of a shift in the peak intensity. This clearly suggests that drying temperature has a significant impact on chemical interactions between GE and SA. However, the addition of EO in the films doesn’t have any significant impact on XRD patterns and this phenomenon is also reported previously in the literature [32,33]. Increase in the drying temperature, improves the crystallinity of edible films (F3–F6, i.e., F4 and F6 are showing more crystalline structure than F3 and F5 which are dried at lower temperatures) [4].

Sodium alginate-based film (F6) dried at 45 °C showed the widest peak, followed by F5, which has a similar composition but dried at a lower temperature, 25 °C. On the other hand, blank film (F1), and films (F3 and F4) without sodium alginate, dried at different temperatures, reveal no significant difference in peak width and intensity. The increase in crystallinity and peak intensity observed in F6 (compared to F5) may be associated with the higher drying temperature [4]. Angle diffraction at the same position (21.4° of 2θ) in all films (F3–F6) reveals better compatibility of the ingredients and uniform crystallinity of the lattice structures within the film matrix [32].

### 3.5. Film Thickness

The thickness of the films ranged from 37.1 to 51.7 μm (Table 2). As per the findings obtained from Table 2, films that were fabricated at room temperature showed more thickness than films exposed to higher drying temperatures (45 °C). Blank films demonstrated less thickness than composite and GEO-loaded films. Relatively, the addition of sodium alginate significantly enhanced the thickness of the films. The difference in the thickness of the films at room and higher temperatures could be attributed to the decrease in moisture content at higher temperatures [34]. The current results are in agreement with findings obtained previously in the literature [17,35]. Incorporation of GEO micro-droplets into the gelatin-sodium alginate network structure may be a reason for more thickness in the films (F3–F6). The addition of sodium alginate further enhanced the thickness because of its high potential in retaining the moisture content compared to gelatin [4].

### 3.6. Mechanical Properties

Packaging material for food must have favorable mechanical features in order to withstand against stress factors presented by the external environment. Moreover, food packaging materials must be mechanically stronger to tolerate physical and chemical changes that occur in the packed material. As per the findings obtained, significant increases in the TS (MPa) and EB (%) have been observed in F3 to F6 in comparison to blank films (F1 and F2). Furthermore, it was observed that TS (MPa) and EB (%) behavior among all films decreased at higher temperatures. The presence of cracks/pores present in the films (SEM analysis) dried at higher temperatures could be responsible for the decrease in TS. Films dried at lower temperatures exhibited better morphology (fewer pores/cracks), which could be associated with an increase in tensile strength. On the other hand, Ym (Mpa) decreased with the incorporation of GEO and SA. Moreover, it was found that Ym values increased with an increase in the drying temperature. An increase in TS and EB values among composite films could be associated with PEC formation, which leads to an increase in inter-molecular hydrogen bonding between GE and SA functional groups. The decrease in TS and EB values at higher drying temperatures could be due to the evaporation of moisture content or a decrease in the inter/intra molecular interactions between the polymers and the GEO that could diminish the homogenous compact structure of the film matrix. In addition, glycerol could also be responsible for changing the mechanical properties of the films [36]. It was also observed that incorporation of GEO improved TS and EB values. This behavior could be due to the increase in the interaction between the polyphenolic components of GEO and polymers [37]. Ym values increased with increasing temperatures among all the films. Furthermore, the addition of GEO and SA decreased Ym values. This could be due to the evaporation of moisture content and GEO at higher temperature which made the films more stiff and rigid than others. Additionally, it was found that incorporation of SA and GEO decreased Ym values, which could be due to the formation of PEC and an increase in molecular interaction among the GEO and polymers [38].

### 3.7. Water Vapor Permeability (WVP)

The water vapor permeability of the films is generally determined by their composition. The hydrophobic components can effectively hinder the transmission of water from the environment to the packed food, which decreases food spoilage. Ideally, lower WVP values are favorable for food packaging materials to avoid any water vapor transmission from the external environment to packed food. As per the results mentioned in Table 2, WVP values are relatively higher in blank films than in others (F3–F6). Moreover, WVP showed higher values for the films developed at higher temperatures than at room temperature. It was also found that SA and GEO addition significantly decreased WVP values (Table 2). As the films (F2, F4, and F6) fabricated at higher drying temperatures showed more cracks and pores, there would be more chances of water transmission from these deformities. The WVP values are higher in the blank film because of the increase in inter-chain spacing among the polymeric chains. The addition of SA possibly reduces this spacing via the formation of PEC. Moreover, incorporation of GEOs reduced WVP values because of the hydrophobic nature of GEOs. However, a previous report in the literature suggested that GEO components have also been reported for their effect in modifying structural configuration via reducing the mass and improving the transmission of WVP through EFs [39]. This report is contradictory to the findings obtained in the present work. However, stable PEC formation between SA and GE may have restricted GEO to impede with properties of the polyelectrolyte complex, in addition to promoting nonpolar nature, resulting in a reduction in WVP transmission [40].

### 3.8. Oxygen Barrier Property (OB)

Ideally, the oxygen permeability of food packaging materials must be less to preserve food from detrimental oxidation reactions [41]. The permeability of films to oxygen depends on several factors, such as their morphology (especially compactness, pores/cracks), crystallinity, and concentration of the EO [42]. Generally, an increase in crystallinity and oil concentration decreases the O2 permeability. However, the presence of defects such as pores increases oxygen permeability. As mentioned in Table 2, films dried at high temperatures showed more oxygen permeability because of the presence of less GEO and more cracks/pores in these films. On the contrary, films dried at low temperatures show less oxygen permeability because of the presence of a high concentration of EO and less pores/cracks in the films. This could be due to volatilization of GEO in films dried at higher temperatures, resulting in the formation of pores, leading to an increase in oxygen permeability. Moreover, it could be due to high chemical interaction between the film matrix and the GEO at 25 °C that results in a compact structure, which may make it more challenging for oxygen molecules (non-polar) to move into the film [43,44]. Thus, our findings suggested that films dried at 25 °C retained more GEO and hence showed more oxygen barrier capacity than films dried at 45 °C.

### 3.9. Swelling Degree (SD) and Water Solubility (WS)

The stability of the edible films is significantly influenced by the hydrophilicity of the components present in the film. Less hydrophilic films offer more resistance to water than more hydrophilic ones. However, natural polymers, which are used in the fabrication of edible films, show more water susceptibility. It was found that water solubility of films prepared at higher temperatures was less than the films prepared at room temperature (Table 3). Moreover, it was noticed that incorporation of GEO and SA decreased solubility. The decrease in the water solubility at higher drying temperatures could be attributed to the increase in the molecular interaction as well as cross-linking between the components. Furthermore, there is a reduction in water solubility after the incorporation of GEO and SA because of their non-polar nature and PEC formation. Additionally, because of their apposite charge, these two polymers form stable PEC via hydrogen bonding and therefore reduce the accessibility of free hydroxyl groups over the film surface. Moreover, GEO components can affect the interaction between -OH of the plasticizer as well as the polymers with water molecules. This behavior led to the increase in water resistance [45].

Swelling is an unfavorable property for an edible film, particularly if meant for packaging of food with a high moisture level [36]. In comparison to the blank (F1, F2), SD values of F3–F6 decreased significantly (*p* < 0.05). However, SD values of the films that were made at higher temperatures were less than those that were made at room temperature. This decrease in SD due to the addition of SA and GEO could be due to the formation of PEC between two polymers. It may also be possible to absorb water due to interference initiated by GEO components with plasticizer behavior. This molecular interaction between the two polymers as well as GEO decreased the interaction with the water and reduced the ability of the EFs to retain water.

### 3.10. Moisture Content (MC)

EFs fabricated for food applications must be preferably resistant to water. However, films made from natural polymers usually have poor water-resistant properties. Thus, to improve the water-resistant property, it is always recommended to add hydrophobic components or to change the architecture of the films by using approaches such as making composite EFs. The MC values of the fabricated films are mentioned in Table 3.

The MC of blank films (F1, F2) were discovered to be greater than that of F3–F6. It was found that the retention of moisture content at higher temperatures decreased considerably. We found no impact of ethanol treatment (70%) on the physical parameters of EFs, i.e., MC. Additionally, incorporation of GEO and SA reduced the retention of moisture content in the films. Decreased MC values of the composite films (F3–F6) could be due to the presence of GEO (non-polar), which may have prevented water transmission into the films. In addition, the decrease in MC may also be due to an increase in the cross-linking between the film components, which has minimized the room for entrapment of more MC [36].

### 3.11. Optical Transmittance Analysis

Transparency as well as color are the important attributes of the films which determine optical properties, appearance, as well as end consumer acceptability. The transparency of the films dried at a lower temperature are comparatively less than films dried at a higher temperature. Additionally, the transparency of the GEO loaded films was found to be less than blank gelatin films (F1, F2). The addition of SA along with GEO to the films (F5, F6) decreased the transparency. Decrease in transparency of the films dried at lower temperatures could be attributed to the presence of a relatively higher moisture content in the films. The decrease in transparency after the addition of GEO and SA could be possibly due to the presence of colorful components in GEO and PEC formation which make the film structure more compact and denser. These structural changes can further interfere with the optical properties of the films. It is optimal to have less or optimal transmittance of the packaging material to provide protection to the packed food against harmful UV radiation [45].

### 3.12. Antioxidant Assays

#### 3.12.1. DPPH Assay

Food oxidation is detrimental in terms of affecting food quality and safety. Oxidation of the food components usually changes its chemical, sensory and nutritional properties. Enzymatic browning, lipid peroxidation, and many others have been reported as deteriorating oxidation processes that considerably impact food quality. Due to their high stability as well as performance, cost-effectiveness, and wide availability, the utilization of synthetic antioxidants is always preferred to reduce or prevent these oxidative reactions in food. The most commonly used synthetic antioxidants are butylated hydroxyanisole (BHA), butylated hydroxytoluene (BHT), propyl gallate, and tert-butyl hydroquinone [46]. However, due to their safety concerns, mainly due to the mounting evidence that draws a relationship between long-term intake of synthetic antioxidants and ill effects on health, natural antioxidants are more preferred these days. Natural antioxidants used in the food industry should be authorized by governing bodies (generally recognized as safe level). These antioxidants must not impact the sensory, chemical, or physical properties of food negatively. Due to the presence of high levels of phenolic components and a safety profile, essential oils have been considered excellent sources of natural antioxidants [47].

**Figure 5:**
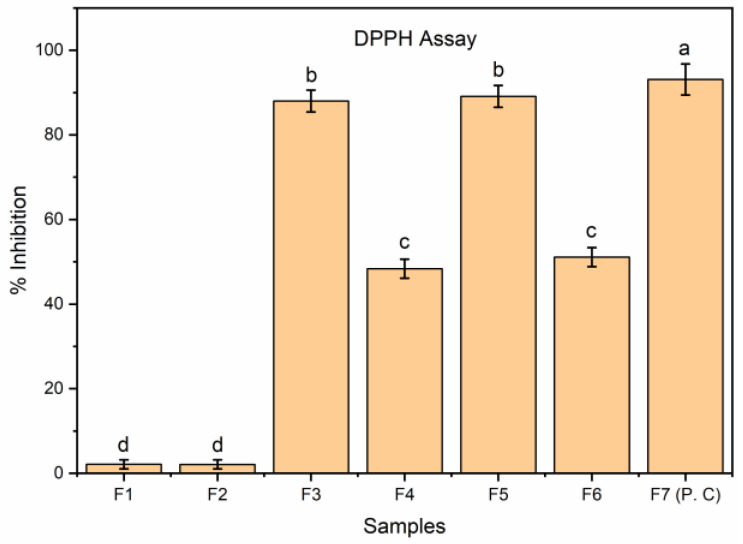
Antioxidant potency (DPPH) of GE-Gly blank films (F1, F2), GE-Gly-GEO films (F3, F4), GE-SA-Gly-GEO film (F5, F6), and butylated hydroxytoluene (F7) determined by DPPH method. Bars correspond to mean values ± standard variations. Bar with distinct characters (a–d) reveals substantial variation between the groups (*p* < 0.05).

Previous work in the literature suggested that rosemary as well as sage essential oils demonstrated comparable antioxidant effects to BHT [48]. EOs can be loaded into EFs to overcome safety concerns associated with synthetic antioxidants. In the present work, GEO was incorporated into GE-SA composite polymeric matrix to primarily study the effect of different drying temperatures of the films and to improve shelf life as well as prevent oxidation of food products. Findings obtained from the Folin–Ciocalteu method showed that GEO had (5.87 ± 0.07) mg of gallic acid equivalents/g of dry mass of the sample of total phenols. This value is close to the value obtained in our previous work [9]. Results obtained from the DPPH assay demonstrated that GE blank films showed the least antioxidant effects, which contradicts with earlier antioxidant effects of gelatin. This could be due to the type of the assay or conditions used in previous work as well as source of gelatin, and type of gelatin hydrolysates [49]. Figure 5 shows the effects of different drying temperatures on the antioxidant effects of EFs. It was found that GEO loaded films prepared at room temperature showed comparable antioxidant effects to butylated hydroxytoluene. However, EFs prepared at high drying temperatures showed comparatively less antioxidant effects. This could be due to the detrimental effect of the high drying temperature on GEO. Thus, at higher drying temperatures, there could be a chemical transformation or evaporation of GEO, which could account for the partial loss of F4 and F6 antioxidant effects. This is in agreement with the previous report [12]. Thus, current findings show that room temperature drying is favorable for retaining antioxidant effects of GEO, as these conditions possibly prevent the chemical transformation as well as loss of EO due to evaporation.

#### 3.12.2. ABTS Assay

**Figure 6:**
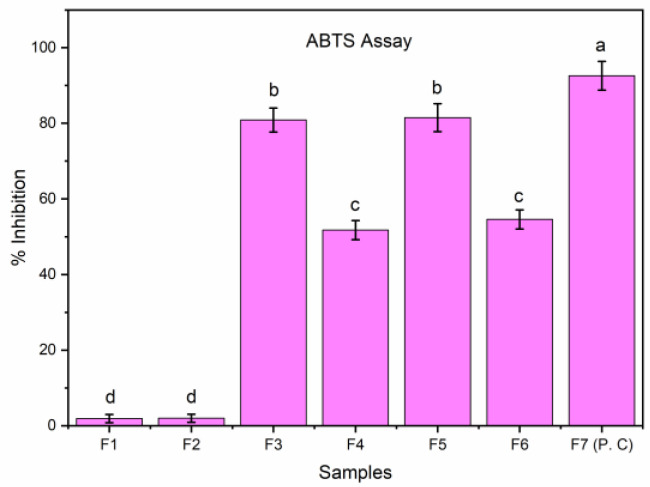
Antioxidant potency (ABTS) of GE-Gly blank films (F1, F2), GE-Gly-GEO films (F3, F4), GE-SA-Gly-GEO film (F5, F6), and butylated hydroxytoluene (F7). Bars correspond to mean values ± standard variations. Bar with distinct characters (a–d) reveals substantial variation between the groups (*p* < 0.05).

In the ABTS assay, GE blank films, dried at different temperatures, exhibited the least antioxidant potency, while the GEO loaded films (F3 and F5) dried at lower temperatures exhibited higher antioxidant potential compared to the positive control (P.C.), butylated hydroxytoluene (Figure 6). Antioxidant activity of films dried at higher temperatures was found to be comparatively less (F4 and F6). A decrease in antioxidant potential in these films could be due to evaporation GEO. The current findings are in accordance with the previous study [12].

## 4. Conclusions

The use of edible films holds key advantages over other commonly used films for food packaging. Edible films are biodegradable and protect food from spoilage by preventing food from contaminating pathogens, reducing oxidation as well as providing UV protection. Gelatin-sodium alginate-based films loaded with ginger oil hold great potential to be used as a new eco-friendly and more potent biologically active edible film. In the current work, results showed that GE-SA-GEO edible films dried at 25 °C presented more favorable surface morphology (homogenous surface and more compact structure with no cracks), mechanical as well as barrier properties (low WVP, favorable thickness, EB, TS, and Ym), and withstand heat when compared to other edible films synthesized at different conditions. This was confirmed by SEM, TGA, FTIR, and XRD analysis. Additionally, GEO-loaded films showed less transparency than other films. Moreover, it was also found that the inclusion of ginger oil confers strong antioxidant potential to GE-SA based edible films.

## Figures and Tables

**Table 1:** Composition of the edible films.

Composition	Drying Temperature	
	25 °C	45 °C
GE (3% *w*/*v*) + Gly (1.5% *w*/*v*)	F1	F2
GE (3% *w*/*v*) + Gly (1.5% *w*/*v*) + T80 (1%) + GEO (1%)	F3	F4
GE (3% *w*/*v*) + SA (3% *w*/*v*) + Gly (1.5% *w*/*v*) + T80 (1%) + GEO (1%)	F5	F6

GE stands for gelatin, Gly stands for glycerol, T80 stands for Tween 80, GEO stands for ginger essential oil, and SA stands for sodium alginate.

**Table 2:** Mechanical properties WVP, oxygen barrier property (OB), thickness, EB, TS, and Young’s modulus (Ym) of the EFs.

Formulations	WVP (×10^−12^ G cm/cm^2^ s Pa)	OB (g/100 g)	Thickness (μm)	EB (%)	TS (MPa)	Ym (MPa)
F1	2.9 ± 0.02 ^c^	3.11 ± 0.013 ^a^	41.12 ± 1.2 ^c^	43.22 ± 8.14 ^d^	28.21 ± 1.36 ^e^	77.21 ± 1.67 ^b^
F2	3.9 ± 0.01 ^a^	3.39 ± 0.043 ^a^	37.11 ± 2.4 ^d^	36.34 ± 6.11 ^e^	19.17 ± 27.1 ^f^	82.11 ± 3.11 ^a^
F3	1.8 ± 0.02 ^d^	2.34 ± 0.059 ^b^	46.22 ± 1.9 ^b^	73.11 ± 4.26 ^b^	55.37 ± 4.28 ^b^	52.55 ± 6.18 ^d^
F4	3.1 ± 0.03 ^b^	3.19 ± 0.049 ^a^	39.11± 2.3 ^d^	47.11 ± 7.21 ^d^	41.32 ± 1.24 ^d^	67.89. ± 2.81 ^c^
F5	1.1 ± 0.02 ^d^	2.15 ± 0.031 ^b^	51.77 ± 1.8 ^a^	88.24 ± 7.14 ^a^	68.11 ± 3.24 ^a^	31.47 ± 2.14 ^f^
F6	2.6 ± 0.01 ^c^	3.26 ± 0.047 ^a^	45.23 ± 1.1 ^b^	53.26± 4.11 ^c^	48.16 ± 6.12 ^c^	48.22 ± 3.18 ^e^

Findings are presented as mean value ± standard deviation. Values with several superscript symbols in each row indicate significant variations among the EFs (*p* < 0.05). WVP: water vapor permeability, EB: elongation at break, TS: tensile strength, Ym: Young’s modulus.

**Table 3:** WS, SD, MC, and transparency of the edible films.

Codes of the Samples	WS (%)	SD (%)	MC (%)	Transparency
F1	41.2 ± 1.2 ^a^	187 ± 15.1 ^a^	28.17 ± 0.14 ^a^	35.11 ± 1.25 ^b^
F2	34.1 ± 0.6 ^b^	95 ± 17.6 ^c^	18.32 ± 0.21 ^c^	48.17 ± 2.15 ^a^
F3	27.2 ± 0.3 ^c^	128 ± 11.4 ^b^	23.12 ± 0.25 ^b^	31.42 ± 1.42 ^c^
F4	18.1 ± 3.2 ^d^	82 ± 6.1 ^d^	19.27 ± 0.16 ^c^	46.32 ± 2.11 ^a^
F5	21.1 ± 1.2 ^e^	85 ± 8.1 ^d^	15.31 ± 0.23 ^d^	15.21 ± 0.31 ^e^
F6	16.4 ± 0.1 ^d^	54 ± 2.8 ^e^	10.12 ± 0.37 ^e^	22.17 ± 0.11 ^d^

All the findings are represented as mean value ± standard deviation. Superscript in every row indicate considerable differences among the mean values of the samples (*p* < 0.05). WS; Water solubility, SD; Swelling degree, and MC; Moisture content.

## Abbreviations

Gelatin (GE), Sodium alginate (SA), Glycerol (Gly), Tween 80 (T80) and Ginger oil (GEO), Polyelectrolyte complex (PEC), Thermogravimetric analysis (TGA), Fourier-transform Infrared spectroscopy (FTIR), X-ray diffraction (XRD), Scanning electron microscopy (SEM), Edible films (EFs), Moisture content (MC), Water solubility (S), Swelling degree (SD), Water vapor permeability (WVP), Young modulus (Ym) Oxygen permeability (OP), Generally recognized as safe (GRAS), Food and Drug Administration (FDA), 2,2-diphenyl-1-picrylhydrazyl (DPPH), Butylated hydroxyanisole (BHA), and butylated hydroxytoluene (BHT).
